# UVB mutagenesis differs in *Nras*- and *Braf*-mutant mouse models of melanoma

**DOI:** 10.26508/lsa.202101135

**Published:** 2021-07-01

**Authors:** Robert L Bowman, Rebecca C Hennessey, Tirzah J Weiss, David A Tallman, Emma R Crawford, Brandon M Murphy, Amy Webb, Souhui Zhang, Krista MD La Perle, Craig J Burd, Ross L Levine, A Hunter Shain, Christin E Burd

**Affiliations:** 1 Human Oncology and Pathogenesis Program, Memorial Sloan Kettering Cancer Center, New York, NY, USA; 2 Department of Cancer Biology and Genetics, The Ohio State University, Columbus, OH, USA; 3 Department of Molecular Genetics, The Ohio State University, Columbus, OH, USA; 4 Department of Biomedical Informatics, The Ohio State University, Columbus, OH, USA; 5 Department of Veterinary Biosciences, The Ohio State University, Columbus, OH, USA; 6 Department of Dermatology, Helen Diller Family Comprehensive Cancer Center, University of California, San Francisco, San Francisco, CA, USA

## Abstract

*BRAF*-mutant melanomas are more likely than *NRAS*-mutant melanomas to arise in anatomical locations protected from chronic sun damage. We hypothesized that this discrepancy in tumor location is a consequence of the differential sensitivity of *BRAF* and *NRAS*-mutant melanocytes to ultraviolet light (UV)-mediated carcinogenesis. We tested this hypothesis by comparing the mutagenic consequences of a single neonatal, ultraviolet-AI (UVA; 340–400 nm) or ultraviolet-B (UVB; 280–390 nm) exposure in mouse models heterozygous for mutant *Braf* or homozygous for mutant *Nras*. Tumor onset was accelerated by UVB, but not UVA, and the resulting melanomas contained recurrent mutations affecting the RING domain of MAP3K1 and Actin-binding domain of Filamin A. Melanomas from UVB-irradiated, *Braf*-mutant mice averaged twice as many single-nucleotide variants and five times as many dipyrimidine variants than tumors from similarly irradiated *Nras*-mutant mice. A mutational signature discovered in UVB-accelerated tumors mirrored COSMIC signatures associated with human skin cancer and was more prominent in *Braf*- than *Nras*-mutant murine melanomas. These data show that a single UVB exposure yields a greater burden of mutations in murine tumors driven by oncogenic Braf.

## Abstract (precis)

A single, neonatal UVB exposure drives greater UV-signature production in Braf- than Nras-mutant murine melanomas.

## Introduction

The most common genetic subtypes of human melanoma, neuroblastoma RAS viral oncogene homolog (*NRAS*)- and v-Raf murine sarcoma viral oncogene homolog B (*BRAF*)-mutant, are enriched in different anatomical locations. *NRAS*-mutant melanomas preferentially localize to chronically sun-damaged (CSD) skin on the head and neck, whereas *BRAF*-mutant melanomas are more common in areas of intermittent sun exposure (Zhang et al, 2016). Despite the association of *NRAS*-mutant tumors with CSD skin, it is reported that UV signature lesions (C>T and CC>TT) are prevalent in a similar proportion of *NRAS*- and *BRAF*-mutant melanomas (The Cancer Genome Atlas, 2015). These observations led us to speculate that BRAF-mutant melanocytes may acquire a higher burden of mutations than NRAS-mutant melanocytes exposed to a single UV exposure. However, it is difficult to control for differences in lifetime sun exposure among biopsies of human melanomas or nevi.

Genetically engineered mouse models (GEMMs) provide a controlled genetic background in which the genomic and phenotypic effects of UV exposures can be studied. GEMMs encoding a melanocyte-specific *Nras*- or *Braf*-mutation mimic the presence of these mutations in human benign nevi (Roh et al, 2015). Moreover, neonatal or chronic UV treatment accelerates the formation and progression of melanoma in a variety of melanoma GEMMs, consistent with human disease etiology (Mukhopadhyay et al, 2016; Chagani et al, 2017; Hennessey et al, 2017; Pérez-Guijarro et al, 2017; Trucco et al, 2019). Genomic analyses of tumors from UV-treated *Nras* or *Braf*-mutant GEMMs have been reported (Viros et al, 2014; Mukhopadhyay et al, 2016; Trucco et al, 2019). However, no study has directly compared the mutational profiles of *Nras*- and *Braf*-driven mouse melanomas exposed to the same UV dosing scheme. Therefore, a complete understanding of how different oncogenic drivers cooperate with environmental mutagens to promote transformation is lacking.

Here, we used a single-dose UV irradiation scheme to characterize the phenotypic and genomic effects of narrowband UVA (340–400 nm) and broadband UVB (280–390 nm) exposures in *Nras*- and *Braf*-mutant mouse models of melanoma. We exposed these animals to a single dose of UVA or UVB, approximating the amount of energy from each band of the UV spectrum in 40 min of intense sunlight. Then, we monitored the mice for melanoma development. Tumors from these animals were sequenced to gain insight into the mutational consequences of each UV source in *Nras*- and *Braf*-mutant melanocytes.

## Results

### UV exposure alters NRAS- and BRAF-mutant melanomagenesis

**Figure 1.**
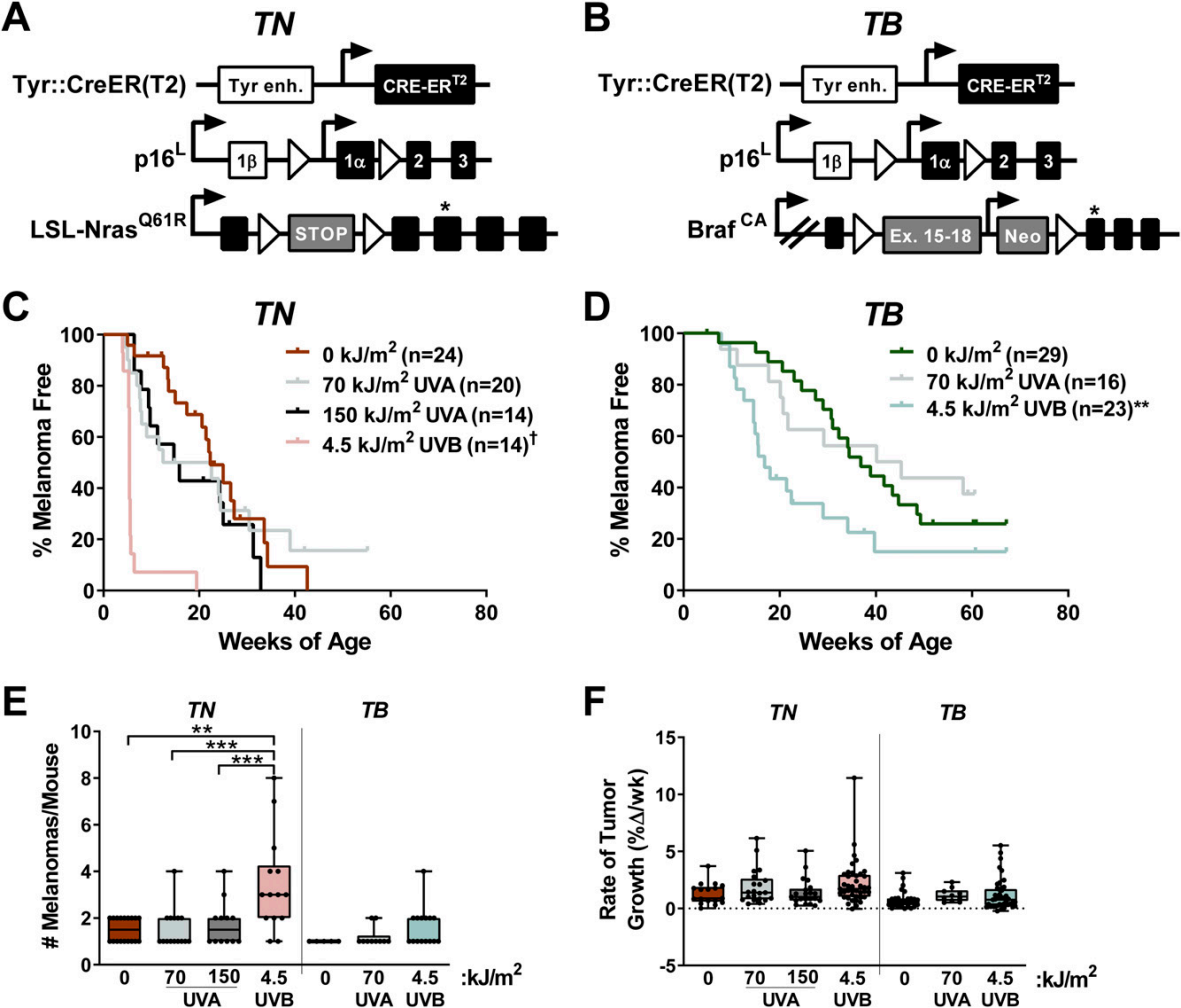
Neonatal UV exposure alters melanoma onset in TN and TB mice. **(A)** *TN* mice are homozygous for a melanocyte-specific, tamoxifen-inducible Cre transgene (Tyr::CreER[T2]), a conditional *p16*^*INK4a*^ knockout allele (*p16*^*L*^), and a conditional *Nras*^*Q61R*^ knock-in allele (*LSL-Nras*^*Q61R*^). Open triangles represent LoxP sites. A star indicates the location of the *Nras*^*Q61R*^ mutation. **(B)** *TB* mice carry a single, *Braf*^*V637E*^ conditional allele (*LSL-Braf*^*CA*^) and are homozygous for Tyr::CreER(T2) and *p16*^*L*^. Note that BRAF^V637E^ is the murine equivalent of human BRAF^V600E^. Open triangles represent LoxP sites and the location of the V637E mutation is indicated by a star. **(C, D)** Kaplan–Meier curves depicting the melanoma-free survival of *TN* (C) and *TB* (D) mice treated on postnatal day three with a single dose of ambient light (0 kJ/m^2^), UVA or UVB. †*P* < 0.0001, **P < 0.01 comparing control (0 kJ/m^2^) and UV-irradiated animals of the same genotype (Gehan–Breslow–Wilcoxon). **(E)** Total tumor burden of control and UV-irradiated *TN* and *TB* mice at euthanasia. Each circle represents a single mouse. Boxes represent the mean and interquartile range for each group. Whiskers span from the minimum to the maximum value. **P < 0.01, ***P < 0.001 comparing control (0 kJ/m^2^) animals of the same genotype (nonparametric ANOVA with Benjamini–Hochberg’s false discovery rate correction). **(F)** Average tumor growth rates for UV- and mock-irradiated *TN* and *TB* mice. Each circle represents a single tumor (*TN*: 0 kJ/m^2^ n = 17; 70 kJ/m^2^ UVA n = 21; 150 kJ/m^2^ UVA n = 21; 4.5 kJ/m^2^ UVB n = 41; *TB*: 0 kJ/m^2^ n = 27; 70 kJ/m^2^ UVA n = 10; 4.5 kJ/m^2^, n = 41). **(E)** Data are presented as described in “(E).”

We generated melanocyte-specific, Tyr::CreER(T2)–driven, *Nras* (*TN*) and *Braf* (*TB*) mice to model the major genetic subtypes of human melanoma (Hodis et al, 2012) (Fig 1A and B). *TN* mice are homozygous for the *LSL-Nras*^*Q61R*^ allele (Burd et al, 2014; Hennessey et al, 2017), whereas *TB* animals carry a heterozygous, conditional *Braf*^*V637E*^ allele (*Braf*^*CA*^; [Dankort et al, 2007]). Notably, the *Braf*^*V637E*^ allele is the murine equivalent of human *Braf*^*V600E*^ (Rad et al, 2013). Oncogene expression is driven by the endogenous gene promoter in both models, and is activated by a melanocyte-specific, tamoxifen-inducible Cre recombinase (Tyr::CreER[T2]; [Bosenberg et al, 2006]). Therefore, the expression of oncogenic *Nras* or *Braf* in these mice mimics the presence of *NRAS* and *BRAF* mutations in most benign human nevi (Roh et al, 2015). Mice carrying only the *Braf*^*CA*^ allele rarely develop melanoma (Dankort et al, 2009). For this reason, *p16*^*INK4a*^ conditional knockout alleles (*p16*^*L*^ [Monahan et al, 2010]) were included in both the *TN* and *TB* models. Although p16^INK4a^ loss-of-function is an early event observed in >60% of human melanomas, germline mutations affecting *p16*^*INK4a*^ are insufficient to drive the disease in mice or humans (Bishop et al, 2000; Shain et al, 2015b; Hennessey et al, 2017).

*TN* and *TB* mice were topically treated with 4-hydroxytamoxifen (4OHT) on postnatal days 1 and 2 to induce Cre activity and stimulate recombination of the conditional *p16*^*INK4a*^ knockout and *LSL-Nras*^*61R*^ or *Braf* ^*CA*^ alleles. On postnatal day 3, the mice were exposed to a single dose of ambient light (“No UV” or 0 kJ/m^2^), narrowband UVA, or broadband UVB irradiation. The amount of UVB delivered approximated that which is contained in 40 min of summer sunlight (4.5 kJ/m^2^), whereas the amount of UVA used models an indoor tanning session (70 or 150 kJ/m^2^; see the Materials and Methods section). These dosing schemes approximate sun exposures of a similar duration, as the UVB to UVA ratio in sunlight is ∼1:20, but varies based on season, cloud cover, and latitude (Cadet & Douki, 2018). Neither dose of UVA or UVB caused erythema or blistering.

**Figure S1.**
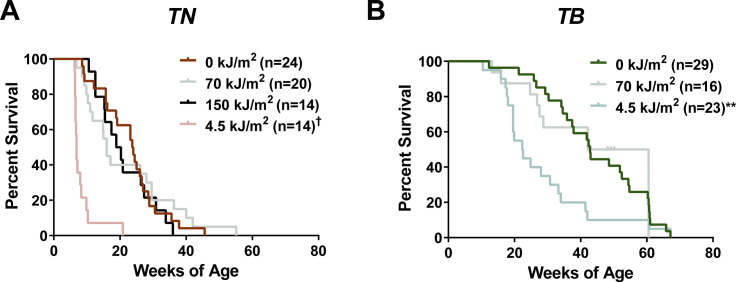
Overall survival of *TN* and *TB* mice treated with No UV, UVA or UVB. **(A, B)** Kaplan-Meier curves depicting the overall survival rates of *TN* and *TB* mice treated on postnatal day three with a single dose of ambient light (0 kJ/m^2^), UVA, or UVB. Mice were euthanized after meeting predetermined exclusion criteria because of tumor burden or malaise. Statistical significance was determined by comparing values from control (0 kJ/m^2^) and UV-treated animals of the same genotype using Gehan–Breslow–Wilcoxon tests. †*P* < 0.0001, **P < 0.01.

The onset of spontaneous melanoma was compared among mice exposed to No UV, UVA, or UVB irradiation. Exposure to a single dose of 4.5 kJ/m^2^ UVB dramatically accelerated melanoma onset and decreased overall survival in both the *TN* and *TB* models (Figs 1C and D and S1A and B). Exposure to 70 kJ/m^2^ UVA led to a modest, but statistically insignificant, reduction in tumor latency as compared with unirradiated controls (avg. MFS = 17.5 and 22.3 wk, respectively; *P* = 0.14; Fig 1C and D). Doubling this dose of UVA in the *TN* model did not further facilitate melanoma formation, suggesting that 70 kJ/m^2^ UVA was sufficient to elicit the maximal response achievable with a single exposure (Fig 1C). Together, these results reveal the potent ability of broadband UVB to promote melanoma formation in *TN* and *TB* mice. Furthermore, our findings suggest that UVA could facilitate melanoma onset in some settings, albeit to a much lesser extent than UVB.

We next examined the incidence and growth phenotypes of tumors arising in each of our experimental cohorts. Total tumor burden (# melanomas/mouse) increased in UVB-exposed *TN* mice but was not significantly altered in *TN* animals treated with UVA or *TB* mice exposed to any form of UV (Fig 1E). Tumor distribution and incidence were also similar between male and female *TN* and *TB* mice regardless of exposure, with ∼59% of tumors arising on the trunk, ∼13% on the head and ∼16% on the ears or tail (data not shown). Once established, *TN* and *TB* tumors grew at the same rate regardless of prior exposure (Fig 1F). Therefore, early tumor onset, rather than more rapid melanoma growth, is responsible for the reduction in overall survival observed in UVB-exposed *TN* and *TB* mice.

**Figure S2.**
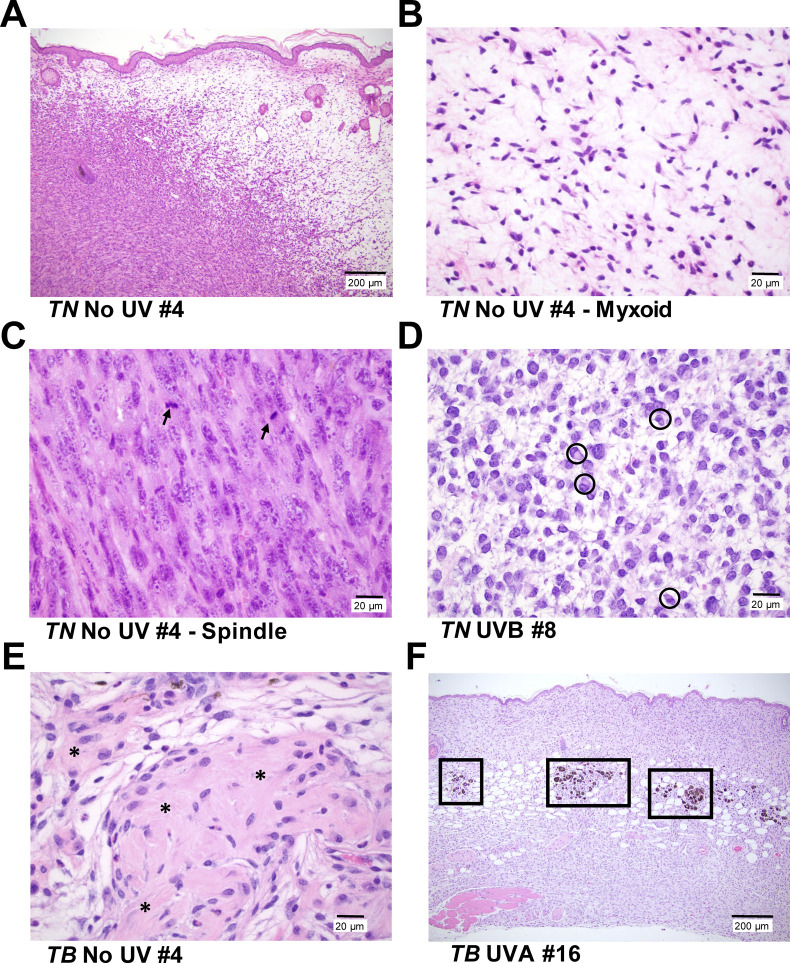
Representative photomicrographs of cutaneous tumors from *TN* and *TB* mice. **(A)** *TN* and *TB* tumors, regardless of UV exposure history, contain variable proportions of spindle (left, cellular) and myxoid (right, spaces) morphologies. Bar = 200 μm. **(B, C)** Higher magnification images of the myxoid (“B,” bar = 20 μm) and spindle (“C,” bar = 20 μm) morphologies depicted in “(A).” Arrows = mitotic figures. **(D)** Representative image of the neoplastic cells with plasmacytoid features seen in most of the *TN* tumors evaluated. (circles; bar = 20 μm). **(E)** Half of the tumors in UVA-treated *TN* mice exhibited a fibroblastic phenotype with abundant collagen (indicated by *; bar = 20 μm). **(F)** Tumor samples from *TB* mice contained areas of pigmentation, typically appearing as clusters of melanophages at the dermal–hypodermal interface (boxes, bar = 200 μm). All images are of hematoxylin and eosin stained tissues.

We postulated that melanomas arising in UVA- or UVB-exposed mice would exhibit distinct histopathological features. Therefore, we examined hematoxylin and eosin stained tumor sections representative of the rate of onset and body site distribution of melanomas from each cohort. Tumors from both models contained variable percentages of myxoid and spindle cells with comparable degrees of invasion, mitosis and granulocyte infiltration regardless of treatment (Fig S2A–C, data not shown). A paucity of pilosebaceous units and hyperplasia of the overlying epidermis was also observed in UVA, UVB, and unexposed mice of both genotypes (Fig S2A and F). Most tumors from the UVB-treated *TN* cohort contained neoplastic cells with plasmacytoid features that were not prevalent in *TN* melanomas from the UVA and No UV cohorts (six of seven versus two of six and zero of five tumors, respectively; Fig S2D). Fibroblastic features were seen in *TN* melanomas from UVA-treated animals (three of six), but were not overtly apparent in tumors from other *TN* mice (Fig S2E). Unlike the *TN* model, tumor samples from *TB* mice contained areas of pigmentation, typically characterized by multiple clusters of melanophages distributed at the dermal–hypodermal interface with or without associated neoplastic cells and occasionally within the tumors (Fig S2F). These results show that although the histopathological features of cutaneous murine and human melanomas differ, a single UVA or UVB exposure can promote the formation of cutaneous, murine tumors with distinct morphologic features.

### Identification of clustered *Flna* and *Map3k1* mutations in *TN* and *TB* melanomas

Prior GEMM studies revealed an enrichment of *Trp53* mutations in melanomas accelerated by full-spectrum (UVA + UVB) or UVB irradiation (Viros et al, 2014; Mukhopadhyay et al, 2016; Trucco et al, 2019). However, *Trp53* mutations occur late in human melanoma pathogenesis (Shain et al, 2018). We sought to identify variants associated with earlier stages of melanoma progression and performed whole exome sequencing using an ensemble calling approach to identify variants in *TN* and *TB* melanomas (see the Materials and Methods section). Pooled normals from each inbred mouse model served as germline controls and polymorphisms observed in dbSNP were excluded (Kitts et al, 2013).

**Figure 2.**
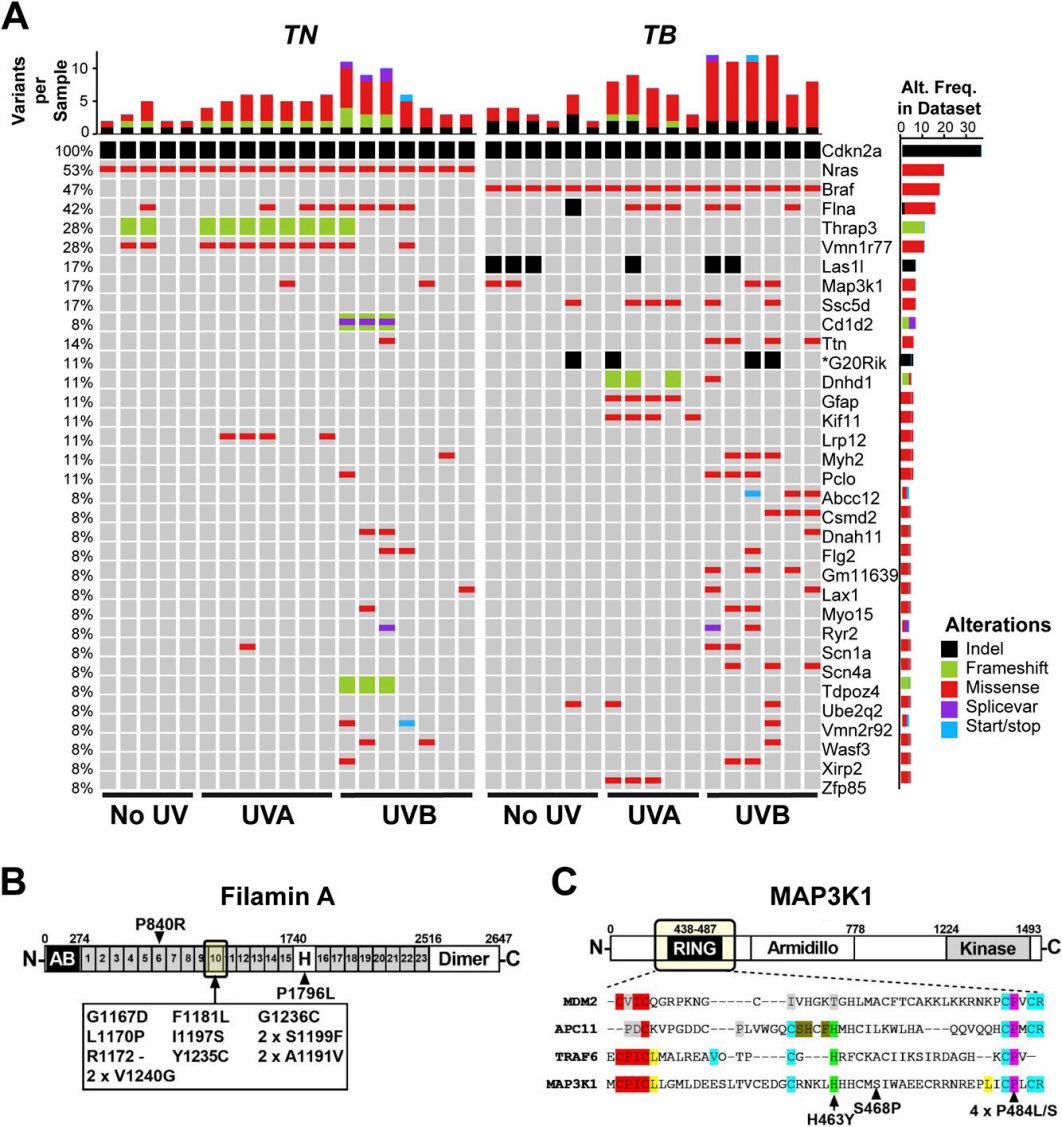
Recurrent genetic alterations in murine models of UV-associated melanomagenesis. **(A)** OncoPrint depicting genes mutated in ≥3 *TN* and *TB* tumors treated with no UV, UVA, or UVB. Color is used to indicate each mutation type: SNV, indel, frameshift, splice variant, or nonsense/stop. Total mutation burden is shown at the top of each sample column. The frequency at which each gene is altered in the dataset is indicated to the right of each row. Visual inception of the raw sequencing data verified *CDKN2a* deletion and *Nras* or *Braf* mutation. **(B)** Schematic depicting the protein domains of Filamin A. A recurrent cluster of mutations was identified in the 10th Ig-like repeat domain as indicated by the arrow. **(C)** Schematic depicting the protein domains of MAP3K1, where recurrent mutations were identified in the RING domain (arrows). Bottom panel depicts a multiple sequence alignment of related E3 ubiquitin ligases, highlighting the conservation of mutated residues.

Unlike previous reports, *Trp53* mutations were extremely rare in melanomas from our models (1 of 36 tumors, Table S1). Therefore, we examined the locations of mutations in any genes found to be altered in three or more of our murine melanomas because prior genomic studies suggest that driver mutations are recurrent and localized. Mutations in *Ttn*, *Gfap*, *Kif11*, and *Vmn1r77* were randomly distributed, suggesting that they are passengers. The only genes we discovered with recurrent, focal mutations were *Flna* and *Map3k1* (Fig 2A–C). Thirteen of the 15 identified *Flna* mutations (87%) localized to the 10th Ig-like repeat of Filamin A (Fig 2B). Alterations in this domain are reported to alter Filamin A binding to F-ACTIN and may also affect protein translation and stability (Nakamura et al, 2007; Page et al, 2011; Suphamungmee et al, 2012). Two *TN* and four *TB* melanomas contained mutations affecting conserved residues of the MAP3K1 RING domain (Fig 2C). These findings are consistent with prior publications implicating MAP3K1 and Filamin A in melanoma progression (Ni et al, 2013; Savoy & Ghosh, 2013; Mann et al, 2015; Trucco et al, 2020).

Table S1 Mutational calls for each tumor shown in Fig 2.

### UVB increases the single-nucleotide variant (SNV) burden of *TN* and *TB* melanomas

**Figure 3.**
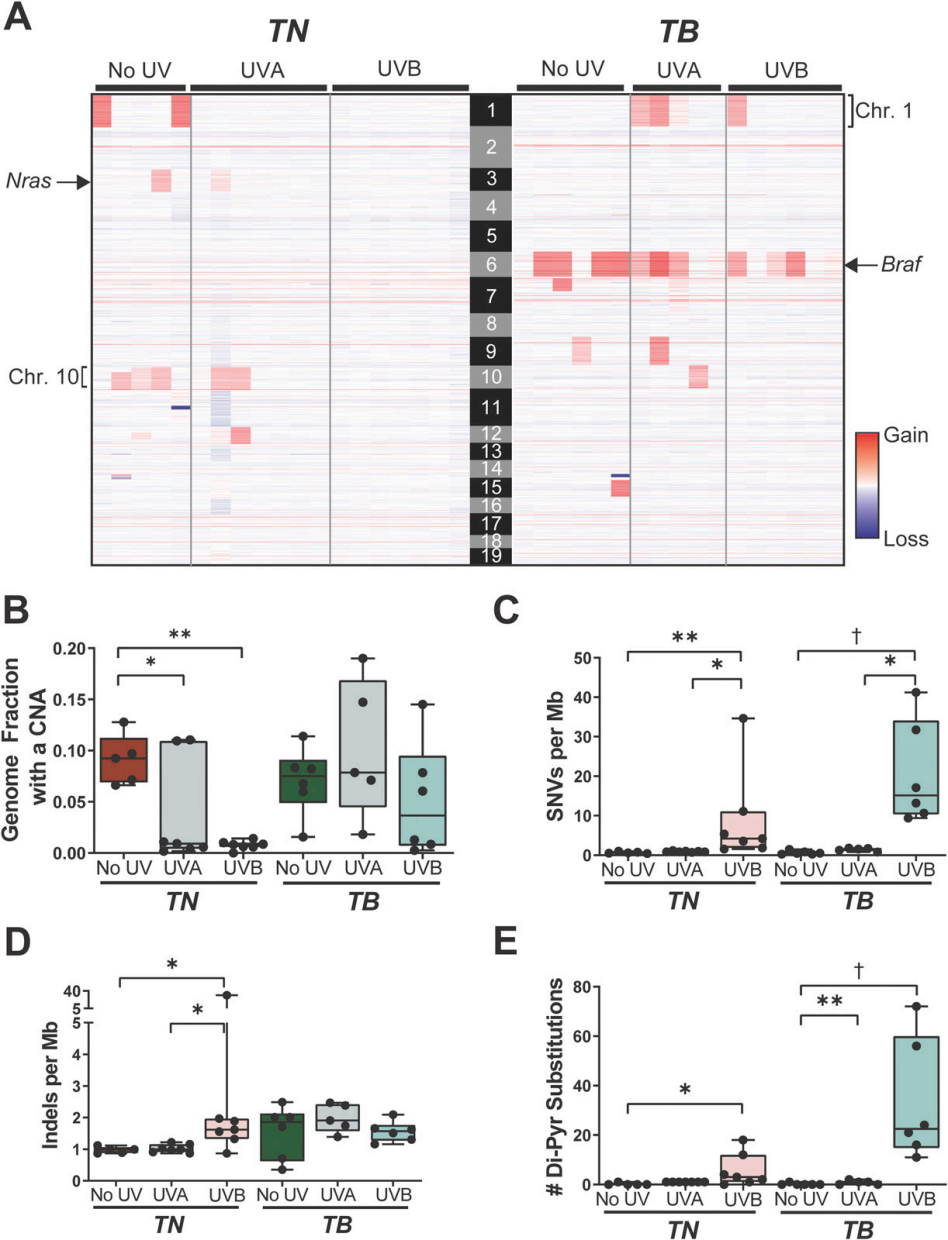
UV alters the genomic landscape of *TN* and *TB* tumors. **(A)** Heat map showing areas of genomic gain or loss within each sequenced tumor. Columns correspond to individual tumors and rows correspond to genomic bins. **(B)** Fraction of each sequenced melanoma genome exhibiting a copy number alteration, graphed as a box plot with whiskers indicating the 5^th^ and 95^th^ percentiles. Dots represent individual tumors. *P*-values determined using nonparametric ANOVA with Benjamini–Hochberg’s false discovery rate correction. **(B, C, D)** Single nucleotide variants (C) and indels (D) per megabase of captured genome, plotted, and analyzed as in “(B).” **(B, E)** Number of dipyrimidine substitutions per tumor, plotted, and analyzed as in “(B).” **(B, C, D, E)** For (B, C, D, E): *P < 0.05, **P < 0.01, ***P < 0.001, †*P* < 0.001.

**Figure S3.**
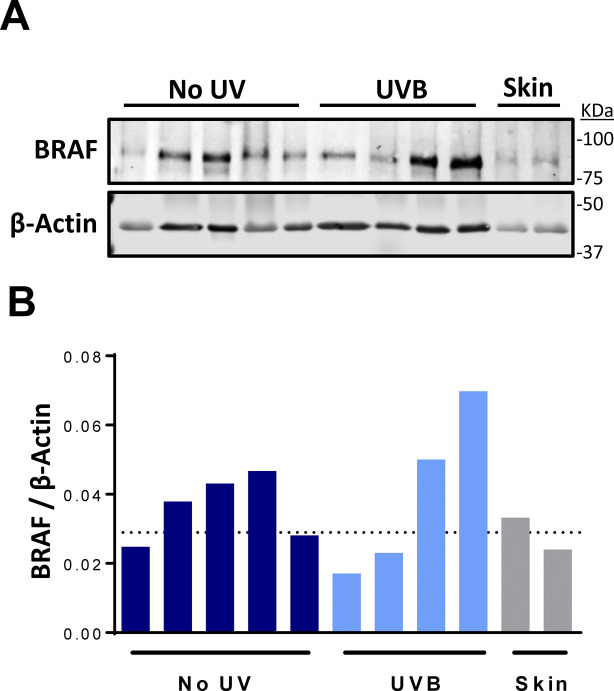
Characterization of BRAF levels in *TB* tumors. **(A)** Immunoblot of BRAF and β-Actin expression in representative *TB* tumors and whole skin. Each row contains lysate from a single tumor or skin lysate. **(A, B)** Quantification of the immunoblot shown in “(A).” The dotted line represents the average Braf expression level in whole skin.

In contrast to human melanomas, tumors from GEMMs are frequently characterized by a high burden of genomic copy number alterations (CNAs) and few SNVs (Hodis et al, 2012; Krauthammer et al, 2012; Zhang et al, 2016; Wang et al, 2017; Zloza et al, 2017). Thus, we sought to determine whether a single UVA or UVB exposure caused significant alterations to the genomic landscape of *TN* or *TB* tumors. Fewer CNAs were seen in all *TN* tumors exposed to UVB and in five of seven *TN* tumors exposed to UVA as compared to melanomas from unirradiated controls (Fig 3A and B). Conversely, only three of six melanomas from our UVB-irradiated *TB* mice had a lower CNA burden than tumors from unirradiated controls (Fig 3A and B). The most common CNA observed in *TB* tumors was a gain in chromosome 6, the chromosome in which *Braf* resides (Fig 3A). Consistent with this observation, 5 of 12 *TB* melanomas showed increased BRAF protein expression as compared to normal, murine skin (Fig S3A and B). These data suggest that there is selective pressure to amplify *BRAF*; however, longitudinal studies are required to test this hypothesis. Recurring copy number gains in chromosomes 1 and 10 were also observed in tumors from the *TB*-UVA, *TN*-No UV, and *TN*-UVA groups (Fig 3A).

The average burden of SNVs increased in both *TN* and *TB* melanomas as a result of prior UVB exposure, whereas the SNV burden of UVA-irradiated *TN* and *TB* tumors was slightly higher, but not statistically different, than that observed in tumors from the No UV groups (Fig 3C; 0.67 versus 0.93 and 1.44 versus 0.65 SNVs/Mb on average, respectively). The frequency of insertions and deletions (indels) did not differ in tumors from irradiated and unirradiated *TB* mice but increased in *TN*-UVB melanomas as compared with unirradiated controls (Fig 3D). Melanomas from UVB-irradiated *TN* and *TB* mice were also enriched for dypyrimidine substitutions, consistent with the ability of UVB to promote cyclobutane pyrimidine dimer (CPD) formation (Fig 3E; [Cadet & Wagner, 2013]).

### UVB drives genotype-dependent mutagenesis on the nontranscribed DNA strand

UVA is the most prevalent form of UV in terrestrial sunlight; however, it is poorly absorbed by DNA (Setlow, 1974; Sutherland & Griffin, 1981; Pfeifer et al, 2005; Khan et al, 2018). By contrast, UVB can directly damage DNA and is the major form of UV responsible for skin erythema and many skin cancers (Setlow, 1974). Both bands of the UV spectrum generate reactive species that promote the formation of a wide variety of modified nucleotides (Cadet & Wagner, 2013). To examine whether distinct mutation types arise after UVA or UVB irradiation, we quantified the burden of each SNV type (C>A, C>G, C>T, T>A, T>C, or T>G) in our sequenced *TN* and *TB* melanomas (Table S2). We also examined the prevalence of C>T transitions at CpG sites because methylated cytosines are reported to form CPDs with higher efficiency than non-methylated cytosines (Tommasi et al, 1997).

**Figure 4.**
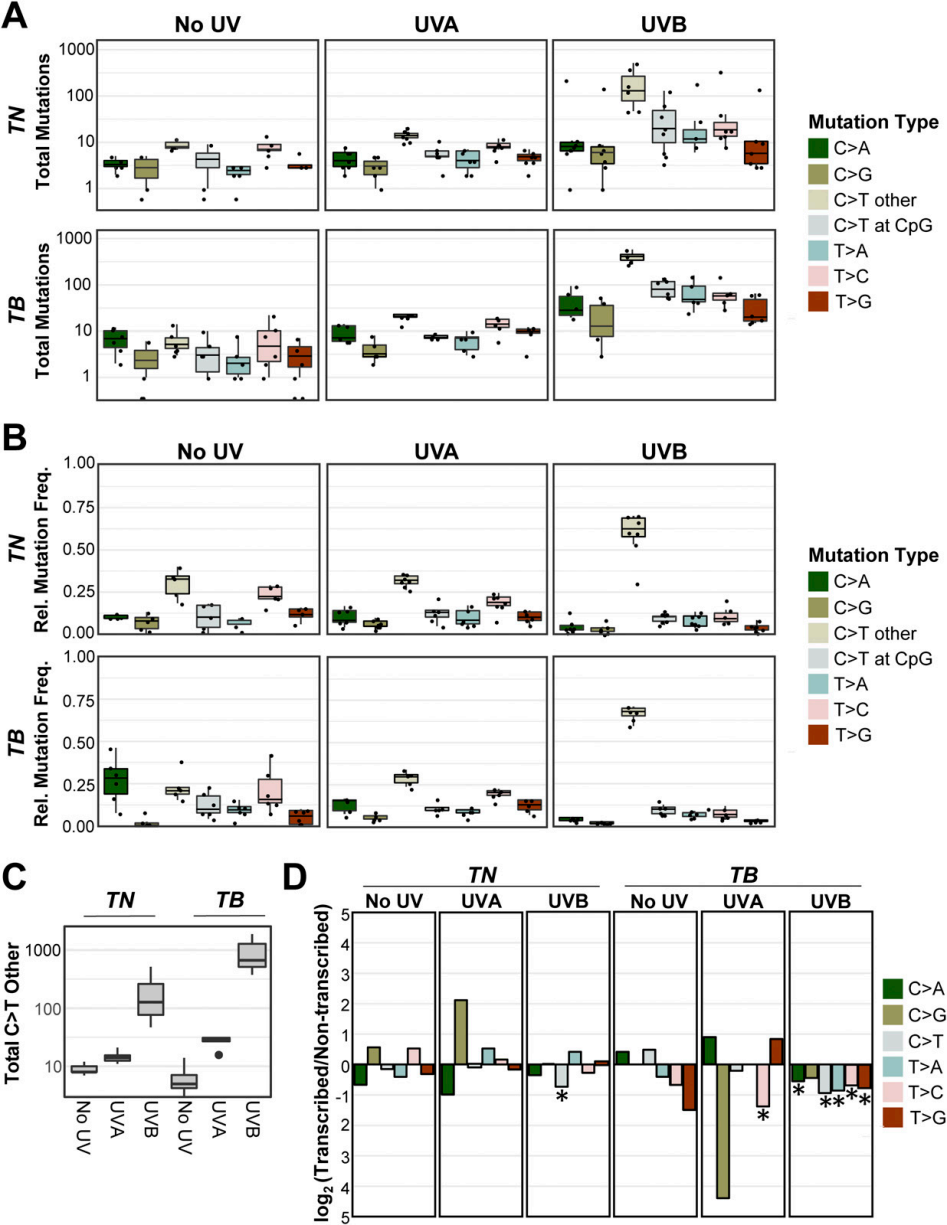
C>T mutations predominate in UVB-induced melanomas. **(A, B)** Absolute mutation burden (A) and frequency (B) of each mutation type in *TN* and *TB* tumors arising after mock (No UV), UVA, or UVB exposure. See Tables S3 and S4 for a complete listing of *P*-values for all comparisons. Statistical significance was evaluated using an ANOVA with Tukey’s HSD post hoc test. **(A, C)** Boxplot as depicted in (A), restricted to only C>T single-nucleotide variants at non-CpG locations. **(D)** The strand location of each mutation type was determined using aggregate data from the indicated mouse models and exposures. Plotted are the log-transformed ratios of transcribed versus non-transcribed mutations. Statistical significance of strand bias was assessed using a Poisson test, where an * indicates significant enrichment. Complete *P*-value listings are found in Table S5.

Table S2 Extended data associated with Fig 4 of the main text.

We compared both the absolute number and relative frequency of each mutation type between tumor types from each genotype and UV irradiation status (Tables S3 and S4). As anticipated, melanomas from UVB-treated *TB* mice had a greater number of C>T transitions than UVA or unirradiated controls of the same genotype (Fig 4A and C and Table S3; *P* < 1.02 × 10^−14^ and 1.82 × 10^−13^, respectively). The absolute number of C>T mutations was slightly, but not significantly, greater in *TN*-UVB than *TN*-No UV tumors (Fig 4A and C and Table S3; *P* = 0.22). However, the relative frequency of C>T mutations in both UVB-irradiated models was greater than UVA or unirradiated tumors of the same genotype (Fig 4B and Table S4; *P* < 3.90 × 10^−3^ for all comparisons). All groups showed a similar number and percentage of C>T alterations at CpG sites, suggesting that methylated cytosines are not preferentially mutated as a result of UVB irradiation (Fig 4B and C). Indeed, differences in C>T burden and frequency were primarily driven by mutations at non-CpG sites (Tables S3 and S4). The increased frequency of C>T mutations was accompanied by decreases in the frequency of T>C mutations in tumors from both UVB-irradiated models (Fig 4B and Table S4). No other mutation types were enriched in a specific genotype or UV irradiation group Tables S3 and S4).

Table S3 (Associated with Fig 4A) Comparison of the absolute mutation burden for each mutation type in *TN* and *TB* tumors arising after mock (No UV), UVA, or UVB exposure.

Table S4 (Associated with Fig 4B) Comparison of the frequency of each mutation type in *TN* and *TB* tumors arising after mock (No UV), UVA, or UVB exposure.

We looked for evidence of oncogene-dependent mutational enrichments and found that C>T transitions were more abundant (*P* < 9.79 × 10^−13^) in *TB*-UVB tumors than *TN*-UVB tumors (Fig 4C and Table S3). Other mutation types did not differ significantly in number or frequency between the two genotypes (Tables S3 and S4). These data highlight differences in the ability of UVA and UVB to drive melanoma-associated mutations and suggest that an underlying *Braf* mutation may promote the accumulation of C>T transitions.

Studies in cultured fibroblasts and model organisms implicate transcription-coupled repair in the rapid repair of UV-induced DNA lesions (Marteijn et al, 2014). Therefore, we investigated whether the SNVs observed in our *TN* and *TB* tumors exhibited a strand bias. Mutations in mock- and UVA-irradiated tumors did not exhibit a strand bias, except in the case of T>C transitions, which were enriched on the non-transcribed strand of *TB*-UVA samples (Fig 4D and Table S5). Tumors from both UVB-treated models showed a bias for C>T mutations on the non-transcribed strand. C>A, T>A, T>C, and T>G mutations were also enriched on the non-transcribed strand of *TB*-UVB, but not *TN*-UVB, tumors. This finding suggests a disparity among *TN* and *TB* melanomas in the biochemistry, incidence, or repair of UV-associated DNA lesions.

Table S5 (Associated with Fig 4D) For each mutation type, a Poisson test was performed to evaluate the statistical significance of the ratio of mutations found on the transcribed and untranscribed strand within the different genotype/UV dosing groups.

### Identification of a UVB mutational signature enriched in *TB*-UVB melanomas

**Figure 5.**
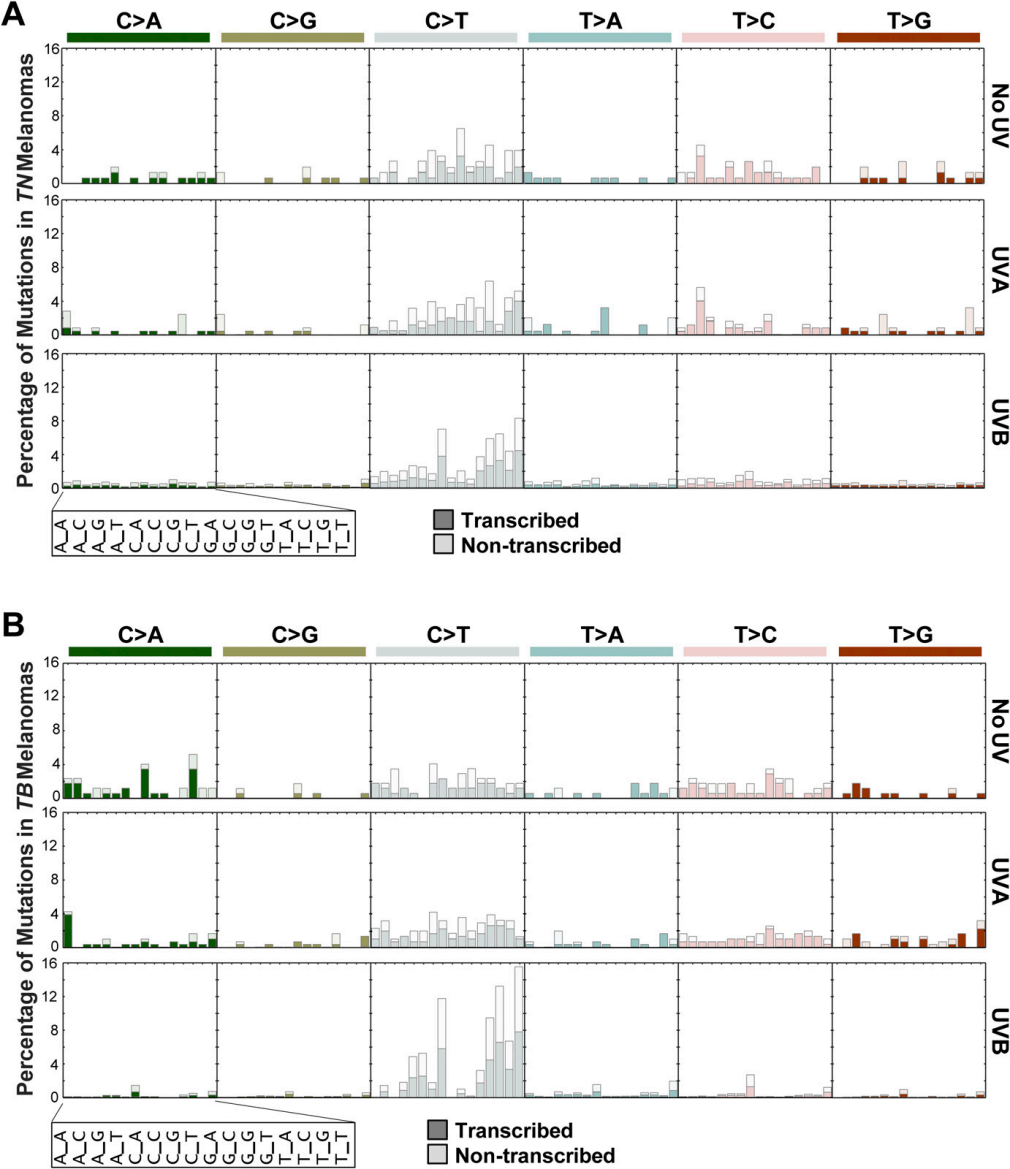
UVB-induced C>T transitions occur within similar trinucleotide contexts in *TN* and *TB* melanomas. **(A, B)** Bar plots indicating the percentage of each indicated mutation type for a given trinucleotide context in *TN* (A) and *TB* (B) tumors. Each subgraph is a mutation type as indicated, where each column within the graph represents a different trinucleotide context surrounding the single-nucleotide variant of interest. Dark shaded bars represent alterations that occur on the transcribed strand, whereas nonshaded bars indicate alterations that occur on the nontranscribed strand. The height of each bar corresponds to the average number of mutations in the indicated experimental group, normalized to the frequency of the relevant trinucleotide sequence in the mouse exome.

CPD-associated C>T lesions are considered classical “UVB signature mutations” and occur preferentially at dipyrimidine sites (Alexandrov et al, 2013). C>T transitions in other, non-cutaneous cancers lack this specificity (Mitchell et al, 1992). For this reason, we took our sequenced *TN* and *TB* melanomas and quantified the burden of each SNV type within every possible trinucleotide context (Table S6). Consistent with these observations, C>T transitions were enriched at TCT and CCT sites in UVB-accelerated *TN* and *TB* melanomas (Fig 5A and B, grey bars). UVB also increased the percentage of C>T mutations at other dipyrimidine sites (CCA, CCC, TCA, TCC, and TCG) as compared with No UV control tumors in the *TB* model. In contrast to UVB-accelerated melanomas, tumors from mock and UVA-irradiated *TB* and *TN* mice showed a similar distribution of mutations amongst the 16 potential trinucleotide sites (Fig 5A and B). These data are consistent with the pattern of C>T mutations previously observed in a Braf-mutant melanoma mouse model chronically irradiated with UVB (Trucco et al, 2019) and prompted us to further explore whether a mutational signature of UVB exposure might be elucidated from our data.

**Figure 6.**
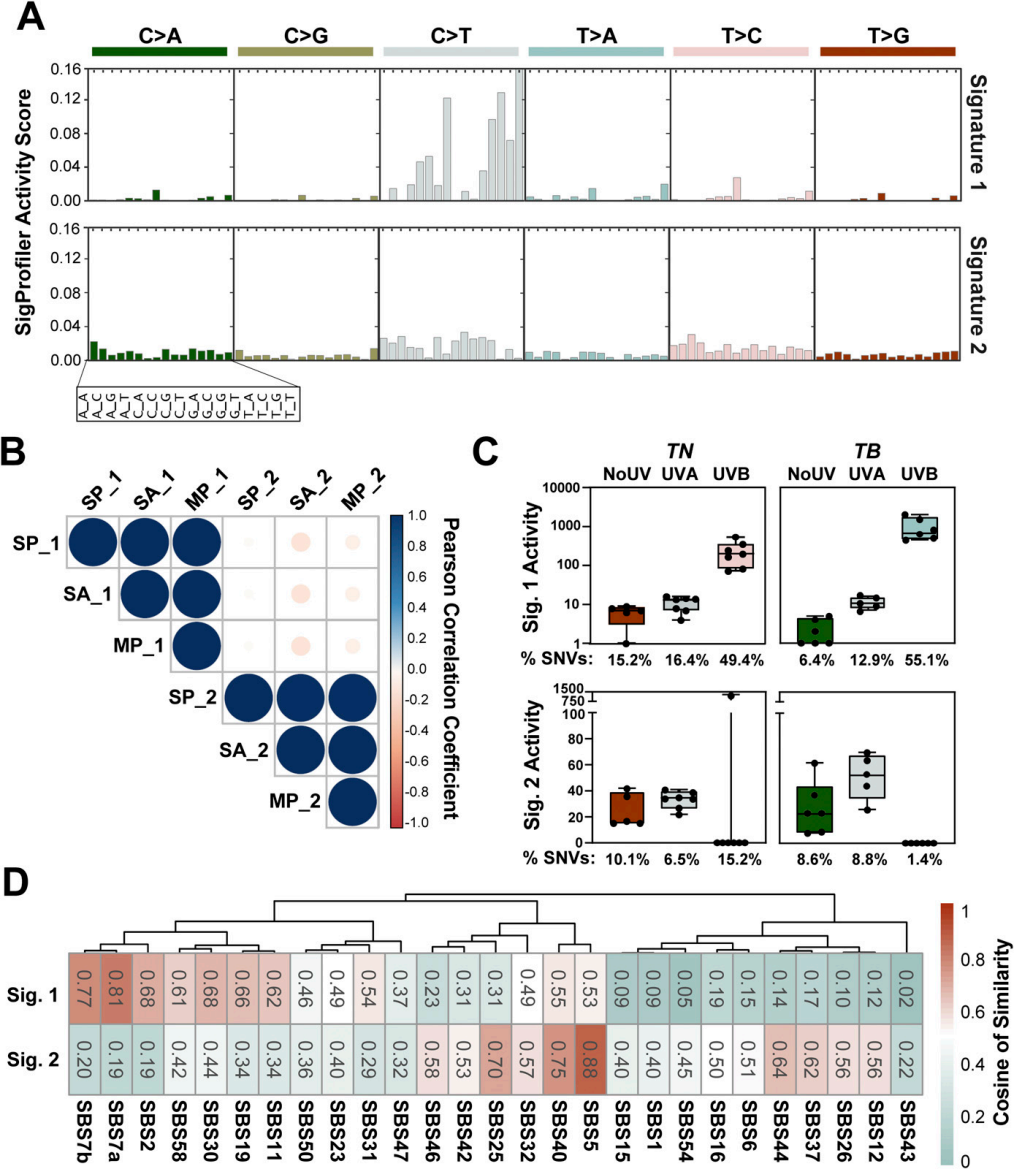
UVB-enriched murine mutational signatures resemble those found in human melanomas. **(A)** SigProfiler was used to identify de novo mutational signatures in sequenced *TN* and *TB* melanomas. Two signatures were selected based on the average Frobenius reconstruction error and signature stability. The y-axis indicates the relative contribution of each trinucleotide mutation type to the discovered mutational signature. Values can be found in Table S7. **(B)** Relationships between signatures derived from SigProfiler (SP_1 and SP_2), MutationalPatterns (MP_1 and MP_2), and SignatureAnalyzer (SA_1 and SA_2) are shown. Color indicates the directionality of each correlation with blue indicating concordance and red indicating discordance. The size of each dot represents the absolute value of the correlation. **(C)** Enrichment of SigProfiler signatures in *TN* and *TB* tumors of each exposure type. Activity scores indicate the number of single-nucleotide variants in each sample attributed to a particular mutational signature. The average percent of single-nucleotide variants represented by each bar is shown below the graphs. Significant differences between groups were assessed using an ANOVA with a Fisher’s least significant difference posttest. Complete *P*-value listings appear in Table S9 and total mutation counts in Table S8. **(D)** Relationship between SigProfiler mutational signatures identified in our dataset (Sig. 1 and Sig. 2) and single base substitution patterns in the COSMIC database. Table S10 contains a complete listing of all *P*-values, which were empirically generated through a cosine similarity permutation test as described in the Materials and Methods.

Table S6 Extended data associated with Fig 6 of the main text.

We used SigProfiler (Alexandrov et al, 2013) to extract co-occurring mutational patterns, “mutational signatures,” from our complete tumor dataset. This method consistently identified two distinct mutational processes operational in our *TN* and *TB* melanomas: Signature 1 and Signature 2 (Fig 6A and Table S7). The profile of Signature 1 contained an abundance of C>T mutations, with a preference for alterations with a 5′ thymidine (TCT>TCC>TCA>TCG, mutated base is underlined). In contrast, the profile of Signature 2 was relatively flat with no specific mutational preference.

Table S7 (Associated with Fig 6A) Fraction of each signature associated with a given mutation and trinucleotide context identified by SignatureProfiler.

**Figure S4.**
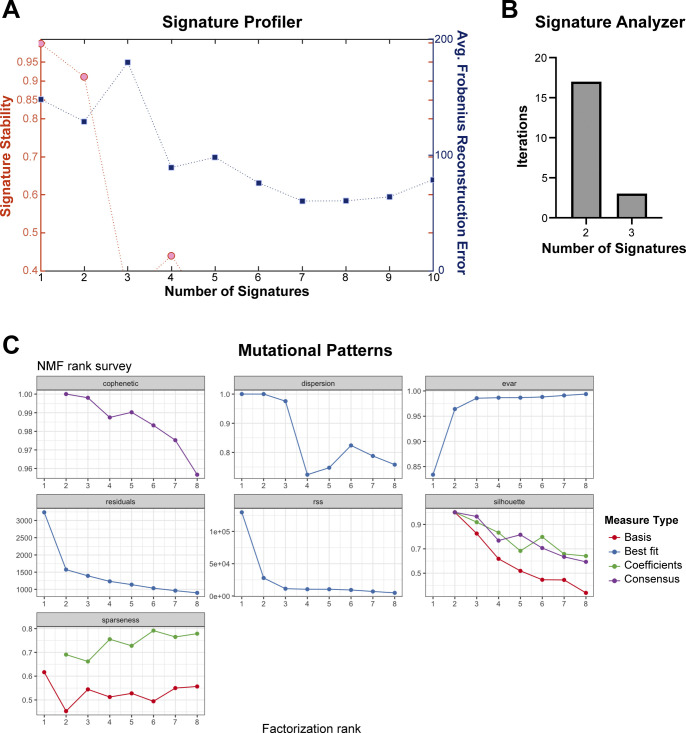
Number of mutational signatures determined by SigProfiler, SignatureAnalyzer, and MutationalPatterns. **(A)** Graph of signature stability and average Frobenius reconstruction error used to determine the number of signatures defined by SigProfiler in the complete melanoma dataset. **(B)** Number of mutational signatures identified in the complete melanoma dataset through 20 iterations of the Bayesian NMF model used by SignatureAnalyzer. **(C)** Results of non-negative matrix factorization using MutationalPatterns. A rank of 2 maximizes the amount of variance explained and the cophenetic score. dispersion = measure of the reproducibility of NMF clusters; evar = explained variance; rss = residual sum of squares; silhouette = NMF measure of consistency within data clusters; sparseness = evaluation of how well the NMF model fits the original data.

The use of multiple extraction algorithms ensures that derived mutational signature is reproducible and robust (Degasperi et al, 2020). Therefore, we used two additional algorithms to extract mutational signatures from our complete dataset: SignatureAnalyzer (Kasar et al, 2015) and MutationalPatterns (Blokzijl et al, 2018). Consistent with SigProfiler, SignatureAnalyzer, and MutationalPatterns identified two distinct mutational processes in our dataset (Fig S4A–C, data not shown). A high degree of similarity was seen between signatures identified by each algorithm, suggesting that the mutational signatures initially found using SigProfiler were robust (Fig 6B).

We next looked to see if either of the SigProfiler (SP) signatures was enriched in a genotype- or treatment-dependent manner in our mouse melanomas. Signature 2 showed a slight enrichment for pyrimidine transitions, but this enrichment was not specific to any genotype or UV treatment, consistent with the idea that Signature 2 represents background noise or mutagenic process common to all experimental groups (Fig 6C). Signature 1 was enriched in melanomas from UVB-irradiated *TN* and *TB* mice over those from No UV- or UVA-treated animals (Fig 6C and Tables S8 and S9). This enrichment was more pronounced in UVB-*TB* than UVB-*TN* tumors, indicating a greater effect of UVB on the mutational profile of *TB* tumors.

Table S8 (Associated with Fig 6C) Total number of mutations attributed to each of the signatures identified with SignatureProfiler.

Table S9 (Associated with Fig 6C) Signature contributions were assessed for the two signatures identified SignatureProfiler.

The enrichment of Signature 1 in UVB-treated mice suggested that this profile could exhibit features in common with mutational signatures enriched in sun-exposed human tumors. Therefore, we looked at the cosine of similarity between the signatures identified in our murine dataset and the catalogue of somatic mutations in cancer (COSMIC) mutational signatures. Our Signature 2 was associated with clock-like signatures of unknown origin that appear to correlate with chronologic age in human tumor datasets, including: SBS40 and SBS5 (Fig 6D and Table S10 [Alexandrov et al, 2013; Alexandrov et al, 2015]). Our Signature 1 closely correlated with SBS7a and 7b (cosine of similarly 0.81 and 0.77, respectively), which are associated with cancers in sun exposed skin and linked to UV damage (Alexandrov et al, 2013). Interestingly, there was no significant association between Signature 1 and two other COSMIC signatures, SBS7c and SBS7d, which are enriched in skin cancer and characterized by T>A and T>C SNVs (cosine of similarity 0.24 and 0.18, respectively) (Alexandrov et al, 2020). These data suggest that mutagenic processes not modeled by our GEMMs may also contribute to human melanomagenesis; however, SBS7a and SBS7b are the predominant signatures found in human melanoma.

Table S10 (Associated with Fig 6C) Relationship between SigProfiler mutational signatures identified in our dataset (Signature 1 and Signature 2) and single base substitution patterns in the COSMIC database.

## Discussion

Human melanoma has one of the highest mutational burdens of any tumor type (Alexandrov et al, 2013). Yet, tumors arising in most melanoma GEMMs, including our unirradiated *TN* and *TB* mice, are largely characterized by CNAs rather than SNVs (Hodis et al, 2012; Krauthammer et al, 2012; Wang et al, 2017; Zhang et al, 2016; Zloza et al, 2017). Here, we find that a single UVB exposure can resolve this conundrum and effectively recapitulate the high burden of SNVs in human melanoma. Furthermore, the pattern of SNVs in our models is representative of mutational signatures observed in the sun-exposed, human tumors (Figs 3E and 6C and D). The UVB-signature derived from our mice does have a higher number of TCT>TTT variants than SBS7a and SBS7b (Fig 6A [Alexandrov et al, 2020]) However, this could be attributed to differences in the trinucleotide frequencies found in each species. Variances in the sequences of transcribed mouse and humans genes could also bias which CPD lesions are efficiently targeted by transcription-coupled repair. Of note, human squamous cell carcinomas deficient in global nucleotide excision repair, exhibit a bias for TCT>TTT variants similar to our models (Chang & Shain, 2020 *Preprint*). Therefore, differences in how human and mouse cells repair UV lesions may explain the increased prevalence of TCT>TTT variants in our UVB signature. The fact that TCT>TTT variants are enriched in other UVB-accelerated GEMMs further supports this hypothesis (Viros et al, 2014; Mukhopadhyay et al, 2016; Trucco et al, 2019).

Our UVB model recapitulates the burden and distribution of SNVs in human melanoma and identifies recurrently mutated genes seen in the human disease. Specifically, we identify clustered *Map3k1* and *Flna* mutations in *TN* and *TB* melanomas irrespective of UV-irradiation status (Fig 2). *Map3k1* is amplified in a subset of human desmoplastic melanomas (Shain et al, 2015a) and was previously linked to melanoma progression by two, independent transposon-mediated mutagenesis screens conducted in the Tyr::CreER(T2) *Braf* ^*CA*^ model (Ni et al, 2013; Mann et al, 2015). Nevertheless, it remains to be determined how mutations affecting the MAP3K1 RING domain influence tumorigenesis. Similar to MAP3K1, functional defects in Filamin A are implicated in the progression of solid tumors (Savoy & Ghosh, 2013). In our models, *Flna* mutations localize primarily to the 10th Ig-like repeat. This domain is responsible for F-actin binding and associated with germline mutations that cause several otopalatodigital spectrum disorders (Moutton et al, 2016). Collectively, these results highlight the potential of forward genetics approaches, like that used here, to offer insights into the distinct evolutionary trajectories initiated by oncogenic and environmental pressures.

Our results indicate a disparity in the melanomagenic potential of UVAI and UVB. Along with prior publications (Noonan et al, 2012; Trucco et al, 2019), these data provide additional evidence that UVA exposures may increase melanoma risk, but not to the same extent as UVB. It is noteworthy that the higher burden of mutations in our UVB tumors did not provide a growth advantage (Figs 1F and 3C). Rather, UVB seems to enhance the ability of genetically predisposed melanocytes to initiate tumor formation. This finding aligns with Blum’s interpretation of the kinetics by which UV initiates non-melanoma skin cancers in albino mice (Blum, 1969). Specifically, Blum hypothesized that UV-dependent tumor initiation requires a combination of genetic and mitogenic effects. Therefore, in our genetically pre-disposed models, UV-induced growth factors may facilitate initial tumor growth leading to an earlier onset.

**Figure S5.**
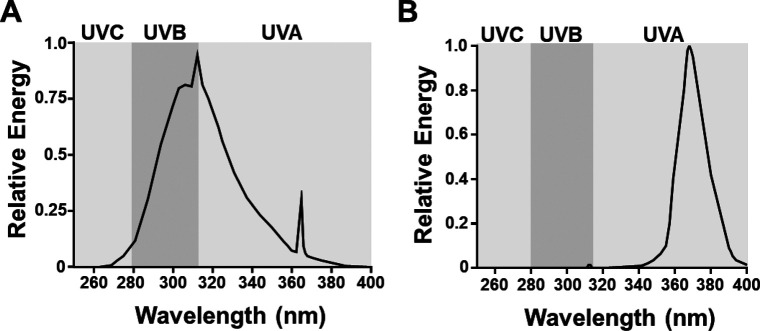
UV light sources. **(A)** Shown is the relative amount of energy produced across the UV spectrum by our narrow band UVB, Spectronics EB-280C light source. **(B)** Shown is the relative amount of energy produced across the UV spectrum by our narrow band UVAI (340–400 nm), BLE-8T365 light source.

We were unable to identify a UVA-specific mutational signature in our samples. However, a 31–44% of SNVs from each tumor were not categorized into Signature 1 or 2. Some of these SNVs may result from weak mutational processes, caused by UVA or other sources, which did not reach the threshold for detection by our extraction algorithms. Because our studies used a narrowband UVA source to avoid bleed-through into the UVB spectrum, we cannot rule out the potential of a broadband UVA source to generate mutational signatures. Furthermore, we cannot be certain that the minor fraction of UVAII remaining in our UVB source does not contribute to Signature 1 (Fig S5). A more granular understanding of the wavelengths responsible for acceleration melanoma onset and driving specific mutational signatures will require more advanced UV filters and light sources than are currently available.

What distinguishes this study from past investigations is the direct comparison of UV carcinogenesis in *Nras*- and *Braf*-mutant melanoma. To establish these highly penetrant models with comparable latencies, we used *TN* mice with homozygous *Nras*^Q61R^ mutations, as heterozygous mice possess a long latency (avg. MFS >45 wk) and low penetrance (<30%). Conversely, *Braf*^CA^ homozygous mice develop rapid disease, precluding the paired zygosity of the *TN* and *TB* models. Although we did not evaluate the response of healthy tissue, this approach did allow us to detect differences in the mutagenicity of a single UVB exposure among melanomas expressing endogenous levels of mutant NRAS or BRAF (Fig 2). Why *TB*-UVB melanomas acquire more mutations than *TN*-UVB melanomas is unclear. One hypothesis might be that BRAF, being downstream of NRAS, requires additional mutations to drive transformation. However, sequencing data show that NRAS-mutant human nevi contain additional driver mutations, whereas BRAF-mutant nevi do not (Shain et al, 2015b). BRAF-mutant human melanomas also have a lower mutational burden than NRAS-mutant melanomas, which is consistent with the fact that NRAS-mutant melanomas are enriched in CSD skin and older individuals, whereas BRAF-mutant melanomas are more common in younger individuals and areas of intermittent sun exposure (Long et al, 2011; Viros et al, 2014; The Cancer Genome Atlas, 2015; Conway et al, 2020). Our data also run contrary to this model in that *TN* and *TB* tumors from our NoUV and UVA cohorts have a similar burden of genomic alterations (*P* adj > 0.33 for all comparisons). Only after UVB exposure do we begin to see genotype-dependent differences in tumor mutational burden.

Another possibility is that Braf-mutant melanocytes are less efficient at resolving UVB-induced DNA lesions. Genotype-dependent DNA-damage responses were recently reported in melanoma cell lines (Sauvaigo et al, 2020 *Preprint*). However, an earlier publication saw no correlation between repair capacity and melanoma genotype (Gaddameedhi et al, 2010). In BRAF-mutant melanomas, loss of p19^ARF^ promotes the epigenetic silencing of XPC, leading to deficiencies in nucleotide excision repair (Luo et al, 2013). Meanwhile, pharmacological inhibition of BRAF has been shown to increase nuclear import of the by-pass polymerase, Pol-K, resulting in increased drug tolerance without clear evidence of enhanced mutagenesis (Temprine et al, 2020). In our mouse models, the differential enrichment of dipyrimidine substitutions and the UVB mutational signature shows that the same UVB exposure ultimately leads to more mutations in BRAF-mutant tumors examined at the time of euthanasia (Figs 3E and 6C).

Finally, BRAF- and NRAS-mutant cells may exhibit differential thresholds for genotoxic stress-induced apoptosis or senescence. This could prevent *TN* melanocytes with a high mutation burden from becoming melanoma. Such a model is not supported by the higher rate of NRAS-mutant melanomas in CSD skin (Long et al, 2011; Viros et al, 2014) but still requires further investigation. In sum, our mouse models establish that the same UVB exposure gives rise to a greater mutational burden in Braf- than Nras-mutant melanomas. These data open the door to further explorations of the underlying causes of oncogene-specific UVB mutagenesis.

## Materials and Methods

### Mouse models

All animal research protocols were approved by The Ohio State University Institutional Animal Care and Use Committee (Protocol #2012A00000134). Mice were backcrossed >7 generations to pigmented, C57BL/6J animals. Heterozygous *LSL*-*BRAF*^CA^ mice were used in these studies, as breeders possessing both BRAF^CA^ and Tyr::CreER(T2) develop both melanoma and non-melanoma tumors at an early age. Inducible knock-in and knockout alleles were activated with 20 mM 4-hydroxytamoxifen on postnatal (p.n.) days one and two as described previously (Burd et al, 2014). Subjects from each litter were randomly assigned to receive either ambient light (No UV), UVA, or UVB on p.n. day 3. A single dose of broadband UVB was delivered to the dorsal side of each animal using a fixed position, 16 W, 312 nm light source (#EB-280C; 280–390 nm; Spectronics). Based upon the spectrum and intensity of this light source, we calculated the McKinlay–Diffey erythemal effective energy (EEE) of a 4.5 kJ/m^2^ dose, delivered over ∼77 s, to be 75 mJ/cm^2^ ([Diffey, 2002]; Fig S5A). A dose of 75 mJ/cm^2^ EEE UVB is equivalent to three human minimal erythema doses in an individual with phototype II skin (i.e., someone who tans minimally, but usually burns with red/blond hair and blue/green/hazel eyes) or to ∼40 min of sun exposure when the UV index is Very High (see Hennessey et al [2017] and Shih et al [2018] for additional information). UVAI (340–400 nm) was similarly delivered using a 16 W source containing two BLE-8T365 bulbs (Spectronics). Based upon the spectrum of these bulbs, the calculated McKinlay–Diffey EEE of a 70 kJ/m^2^ dose is 14.2 mJ/cm^2^ ([Diffey, 2002]; Fig S5B). The average tanning parlor dose is 4.5 Standard Erythema Doses (Dowdy et al, 2011). One SED is equivalent to 10 mJ/cm^2^ EEE-weighted UV light (Diffey, 2002). Approximately half of this exposure derives from the 1–2% of UVB light contained in a tanning bed lamp (UVA dose = 45 mJ/cm^2^/2 or 22.5 mJ/cm^2^). Therefore, an individual receives >1.5 times more UVA in an average tanning session than a mouse in our 70 kJ/m^2^ experimental protocol (22.5/14.2 mJ/cm^2^ = 1.58).

### Tumor monitoring, processing, and histopathology

Mice were randomly numbered following treatment and blindly monitored three times a week for tumor formation. Established melanomas were measured by calipers at least three times per week and tumor size (width × length [mm]) recorded until protocol exclusion criteria were met. Representative tumors were harvested from each cohort, fixed in 10% neutral buffered formalin, routinely processed, and embedded in paraffin wax. Sections (4 μm) were stained with hematoxylin and eosin and evaluated by a veterinary pathologist, certified by the American College of Veterinary Pathologists (KMDL), using an Olympus BX45 light microscope with attached DP25 digital camera (B&B Microscopes Limited).

Tumor morphology was assessed by a certified member of the American College of Veterinary Pathologists (KMDL) using methods described by Banerjee and Harris (2000). In each sample the extent of skin and subcutis tumor invasion, tumor pigmentation and the maximum number of mitotic figures were determined from three different fields of view using a 40× objective and 10× ocular lens with a field number of 22 mm.

### Whole exome sequencing

Tumor DNA was isolated from flash frozen tissue using the Quick-DNA Miniprep Plus Kit (Zymo Research). Tissues were placed in 2-ml tubes containing 190 μl of diluted Zymo Solid Tissue Buffer and 3.0-mm zirconium beads (Cat no. Z763902; Sigma-Aldrich). Samples were then subjected to homogenization using the Precellys Evolution Homogenizer (Bertin Instruments) using the preset elastic setting: speed: 6,800 rpm, cycle: 4 × 30 s, pause: 45 s. Homogenized samples were then incubated in 20 mg/ml of Proteinase K overnight at 55°C before continuing with the Solid Tissues protocol as described for the Quick-DNA Miniprep Plus Kit. Control DNA was generated from toe clips or splenic tissue derived from 10 representative *TN* and 10 representative *TB* animals. These controls were then combined at an equal ratio and concentrated using the Genomic DNA Clean & Concentrator-10 kit (Zymo Research). The integrity and concentration of resulting genomic DNA was confirmed on an Agilent TapeStation.

Indexed libraries were generated from 200 ng of genomic DNA using the Kapa Hyper Prep and Agilent SureSelectXT Mouse All Exon target enrichment systems. Exome hybridization was conducted using 500 ng of each DNA library and the resulting target-enriched fragments were PCR-amplified (11 cycles). Indexed libraries were pooled and subjected to paired-end 150 bp sequencing on an Illumina HiSeq4000. Average target coverage was 75× (range 53–107×). An overview of whole exome sequencing mapping and coverage metrics appears in Table S11.

Table S11 Whole exome sequencing quality, mapping, and coverage metrics by sample.

### Variant calling

Sequences were aligned to mm10 using burrows-wheeler aligner (version 0.7.15) (Li & Durbin, 2009). Duplicates were removed using Picard version 2.17.11 and the resulting sequences re-aligned around indels using GATK version 3.6 (McKenna et al, 2010). Variants were called using VarScan2 (version 2.4) (Koboldt et al, 2012), Mutect2 (Cibulskis et al, 2013), and Strelka2 (Kim et al, 2018). Variants identified by all three callers were filtered to remove existing variations in the Ensembl mouse variation database (Yates et al, 2020) and annotated using Variant Effect Predictor (McLaren et al, 2016). More than 200 calls across samples were visually inspected for depth, alignment, and read quality in Integrated Genomics Viewer (Robinson et al, 2011). Dipyrimidine mutations were counted as a single event when calculating total mutational burden.

### Analysis of SNVs and CNAs

SNV burden (variants/Mb) was calculated as a function of the total capture region. SNVs occurring within a dipyrimidine sites were counted as a single event. Oncoprints of genes mutated in three of more mouse melanomas were made with ComplexHeatmap version 2.0.0 (Gu et al, 2016). To calculate the overlap with human tumors, CNAs were identified using CNVkit (Talevich et al, 2016). Reported CNAs passed a log_2_ segmentation threshold of 0.2 with support from at least five bins. Genome fraction containing a CNA was determined by computing the footprint of segments surpassing the copy number threshold and dividing this by the total footprint of all segments.

### Tumor immunoblotting

Flash-frozen tumors were homogenized in PBS with Halt phosphatase inhibitor (Thermo Fisher Scientific) and protease inhibitor (Sigma-Aldrich) using the Precellys Evolution Homogenizer with Cryolys (Bertin Instruments). The preset elastic setting was used for homogenizing. Tumor homogenates were centrifuged to remove PBS and resuspended in RIPA buffer with phosphatase and protease inhibitors. Lysates were sonicated 2 × 10 s using a Branson digital sonifier at 10% amplitude. Samples were centrifuged at 15,000 rpm for 5 min at 4°C and supernatants collected and quantified by Bradford assay (Bio-Rad). Samples (35 μg total) were blotted for BRAF (sc-5284; 1:500; Santa Cruz) and β-Actin (#3700; 1:1,000; Cell Signaling) and imaged using a LI-COR Odyssey CLx system. Bands were quantified using Image Studio Version 5.2 software (LI-COR Biosciences).

### Mutational spectrum analysis

The total burden and relative contribution of each mutation type to No UV-, UVA-, and UVB-induced melanomas was determined using the “mut_type_occurrences” algorithm in the R package for *MutationalPatterns* (Blokzijl et al, 2018). Differences in the absolute number of mutations were assessed using a Mann–Whitney U test. Differences in frequency of each SNV type between UVA or UVB samples versus controls (No UV) were determined using t-tests with Holm’s adjustment for multiple comparisons (*P* < 0.05 considered significant).

A MATLAB implementation of SigProfiler (Alexandrov, 2020) and an R implementation of SignatureAnalyzer (Kim et al, 2016) were used to identify de novo mutational signatures. Average Frobenius reconstruction error and signature stability were used to select the number of signatures in SigProfiler. The number of signatures selected by SignatureAnalyzer was determined using a Bayesian non-negative matrix factorization (NMF) model described previously (Kim et al, 2016), where two signatures were the most frequent selection from 20 iterations. *MutationalPatterns* was used to examine strand bias and identify de novo mutational signatures (Blokzijl et al, 2018). The number of signatures was selected using non-negative matrix factorization, and a rank of 2 was chosen based on maximization of variance explained and cophenetic score. Comparison of de novo mutational signatures from SigProfiler and those appearing in COSMIC version 3 (Alexandrov et al, 2020) was completed using a cosine of similarity test, for which empirical *P*-values were generated based on 1,000,000 permutations using the “cosinePerm” function from the PharmacoGx package (Smirnov et al, 2016).

## Data Availability

All raw sequencing data generated in this study have been submitted to NCBI Sequence Read Archive (https://www.ncbi.nlm.nih.gov/sra) under accession #PRJNA574176 Code and scripts can be found at https://github.com/bowmanr/UV_mouse_melanoma.

## Supplementary Material

Reviewer comments
